# Zinc supplementation in patients with cirrhosis and hepatic encephalopathy: a systematic review and meta-analysis

**DOI:** 10.1186/s12937-019-0461-3

**Published:** 2019-07-06

**Authors:** Ying-Chi Shen, Ya-Hui Chang, Ching-Ju Fang, Yang-Sheng Lin

**Affiliations:** 1 Department of Medicine, MacKay Medical College, New Taipei City, Taiwan; 2 MacKay Junior College of Medicine, Nursing, and Management, Taipei, Taiwan; 3 Department of Pharmacy, MacKay Memorial Hospital, Taipei, Taiwan; 4 School of Pharmacy, College of Pharmacy, Taipei Medical University, Taipei, Taiwan; 5 Medical Library, National Cheng Kung University, Tainan, Taiwan; 6 Department of Secretariat, National Cheng Kung University Hospital, College of Medicine, National Cheng Kung University, Tainan, Taiwan; 7 Division of Gastroenterology, Department of Internal Medicine, MacKay Memorial Hospital, Taipei, Taiwan; 8 Graduate Institute of Clinical Medicine, College of Medicine, Taipei Medical University, Taipei, Taiwan; 9 Evidence-Based Medicine Center, MacKay Memorial Hospital, Taipei, Taiwan; 10 Division of Gastroenterology and Hepatology, Department of Internal Medicine and Deputy Director, Evidence-Based Medicine Center, MacKay Memorial Hospital, No. 92, Sec. 2, Zhongshan N. Rd., Zhongshan Dist., Taipei City, 104 Taiwan, Republic of China

**Keywords:** Hepatic encephalopathy, Cirrhosis, Zinc, Systematic review

## Abstract

**Background:**

Low serum zinc level is associated with hepatic encephalopathy (HE), but the efficacy of zinc supplementation remains uncertain. This study aimed to investigate the effects of zinc supplementation on HE treatment in patients with cirrhosis.

**Methods:**

We searched MEDLINE, EMBASE, the Cochrane Central Register of Controlled Trials (Cochrane CENTRAL) and Scopus from inception to December 2018; without publication date or language restrictions. Randomized controlled trials of zinc supplementation versus placebo or other treatment for the management of HE in adult patients with cirrhosis were selected. The primary outcome was the degree of HE as assessed by clinical signs or specialized psychometric tests. The secondary outcomes included serum ammonia levels, adverse events, or the length of hospital stay and costs. We carried out a meta-analysis with random effects model and summarized continuous outcomes using standardized mean differences (SMD) or mean differences (MD) with 95% confidence intervals (95% CI). The risk of bias was assessed using the Cochrane risk of bias tool, and the certainty of evidence for each outcome was evaluated with the Grading of Recommendations, Assessment, Development, and Evaluation approach.

**Results:**

Four trials with 247 patients were included. In patients with cirrhosis who had mild HE (≤ grade II), the available evidence suggested that the combination treatment of zinc supplementation and lactulose over 3 to 6 months significantly improved performance in the number connection test (SMD: -0.97; 95% CI: − 1.75 to − 0.19; *P* = 0.01; moderate certainty), reported in three trials (*n* = 227). However, compared with lactulose therapy alone, additional zinc supplementation demonstrated no significant difference in the digit symbol test (SMD: 0.44; 95% CI: − 0.12 to 1.00; *P* = 0.12; very low certainty) or serum ammonia levels (MD: -10.86; 95% CI: − 25.73 to 4.01; *P* = 0.15; very low certainty), reported in two trials (*n* = 137). None of the included trials reported adverse events or effects on hospitalization.

**Conclusions:**

In conclusion, a combination of zinc supplementation and lactulose over 3 to 6 months may improve the number connection test in cirrhotic patients with low grade HE, compared with lactulose only.

**Trial registration:**

PROSPERO: CRD42017080955. Registered 23 November 2017

**Electronic supplementary material:**

The online version of this article (10.1186/s12937-019-0461-3) contains supplementary material, which is available to authorized users.

## Background

Hepatic encephalopathy (HE), which develops in 50–70% of patients with cirrhosis [1], is a serious complication of chronic liver diseases. Based on the severity of manifestation, HE can be categorized as overt HE (OHE) and minimal HE (MHE). OHE, which is graded from I to IV using the West Haven Criteria [2], can be diagnosed by apparent impairment in cognitive or neuromuscular function, while MHE usually requires specialized psychometric or neuropsychological tests for its diagnosis [3]. It is more practical for clinical use, to combine MHE and HE grade I into covert HE (CHE) because of the challenges with diagnoses of these two entities [4].

Although the pathophysiology of HE is not fully understood, hyperammonemia is detected in most patients with HE [5]. Therefore, therapy aimed at ammonia level reduction contributes to HE resolution. Non-absorbable disaccharides, lactulose, and lactitol, which may reduce plasma ammonia levels, are considered to be the standard therapy for episodic OHE [6]. However, despite treatment with disaccharides, HE persists in 20–30% of cirrhotic patients [7, 8]. Several studies [9–11] have shown that nutritional supplementation may be effective for liver cirrhosis. It has been demonstrated that zinc deficiency is common in patients with liver cirrhosis [10–12]. Lower serum zinc level has also been seen as a precipitating factor for HE [12]. Several studies [13–15] investigated the link between zinc and HE, but the overall evidence regarding the effects of zinc therapy for HE remains inconsistent.

A previous meta-analysis conducted by Chavez-Tapia et al. in 2013 [16] included four trials and 233 participants who were diagnosed with cirrhosis and HE. Findings of this previous study indicated that oral zinc supplementation was associated with a significant improvement in performance on the number connection test (NCT) but did not affect HE recurrence. However, Chavez-Tapia et al. compared zinc groups with both placebo and standard lactulose therapy; they also did not identify the MHE in participants in each selected trial. Moreover, a well-designed randomized controlled trials (RCT) [17] which investigated the effectiveness of antioxidants and zinc gluconate on MHE versus lactulose, was published thereafter.

Therefore, we performed an updated systematic review and meta-analysis based on current evidence to estimate the effects of zinc supplementation in patients with cirrhosis and HE.

## Methods

The systematic review was registered in the International Prospective Register of Ongoing Systematic Reviews of the National Institutes of Health Research (CRD42017080955). We performed a systematic review with meta-analysis according to the Preferred Reporting Items for Systematic Reviews and Meta-Analysis (PRISMA) Statement [18] and completed the PRISMA checklist as seen in Additional file 1.

### Data sources and search strategy

A medical librarian (CJ F) at the teaching hospital conducted a comprehensive computerized search of relevant literature in the following electronic databases: MEDLINE, Embase, Cochrane CENTRAL, and Scopus, from inception to December 2018, without publication date or language restrictions. Unpublished articles were identified through searching of the WHO International Clinical Trials Registry Platform (ICTRP). Auto-alerts were established to identify newly released studies. We also hand-searched the reference lists of selected articles to find additional studies. The main keywords used in the search were as follows: hepatic encephalopathy, liver cirrhosis, and zinc, including their controlled vocabularies (MeSH and Emtree terms) and synonyms (text words). Our search terms and strategy were described in Additional file 2.

### Study selection

Studies meeting the following criteria were included: (1) study design: RCTs; (2) population: adults (> 18 years old) with established liver cirrhosis and a history of HE; (3) interventions: oral or parenteral zinc supplementation, regardless of the dose, frequency, or duration; (4) comparators: placebo or other intervention; and (5) primary outcomes: the degree of HE or mental status assessed by clinical signs or specialized psychometric tests [19]; secondary outcomes: serum ammonia levels, adverse events, or hospitalization.

The exclusion criteria were as follows: (1) population: pregnancy, congenital liver diseases or autoimmune liver diseases; (2) studies comparing different doses of the same medication only; and (3) studies without a designated intervention or comparator.

### Data extraction and quality assessment

Two reviewers (YC S and YH C) independently screened the titles and abstracts to identify potentially relevant articles, conducted full-text reviews of eligible studies, performed data extraction, and assessed the quality of each study. Disagreements between reviewers were resolved by discussion with a third reviewer (YS L). The following data were extracted from each selected study: the first author’s name and year of publication, trial design, country, sex, age, etiology of cirrhosis, serum zinc levels, HE grades, Child-Pugh (CP) score or classification, intervention, comparison, and treatment duration. When data were not provided in publications, we attempted contacting the authors for further information.

The methodological quality of the studies was evaluated using the Cochrane Risk of Bias Tool [20]. For each eligible trial, we judged articles as having a low, unclear, or high risk of bias for the following domains: random sequence generation, allocation concealment, blinding of participants and personnel, blinding of outcome assessment, incomplete outcome data, selective reporting, and other sources of bias. For a trial to be categorized as having a low risk of bias, all domains had to be judged as low risk. If at least one domain was classified as unclear or with no high risk of bias domain, the overall risk of bias for the trial was classified as unclear. Similarly, if at least one domain was assessed as having a high risk of bias, the overall risk of bias for the trial was also regarded as high.

### Data synthesis and statistical analysis

Computations for the meta-analysis were conducted using the RevMan 5.3 software (The Nordic Cochrane Centre, The Cochrane Collaboration, 2014). Continuous outcomes were presented as mean differences (MD) if they were measured on the same scale; otherwise, they were presented as standardized mean differences (SMD). Corresponding 95% confidence intervals (95% CI) were calculated for all estimates. We used Hedges’ g score for individual studies as a measure of effect sizes, which was determined by calculating the SMD between groups (SMD: 0.2- < 0.5 = small effect, 0.5- < 0.8 = moderate effect, ≥0.8 = large effect) [21]. In view of the significant heterogeneity, we used the random effects model with the DerSimonian and Laird estimate [22] for pooling. Statistical heterogeneity between studies was assessed using both the chi-squared test and the I^2^ statistics. Either *P* < 0.10 or I^2^ > 50% indicated substantial heterogeneity [23]. We tabulated the summary of the findings and certainty of evidence for each outcome (classification as high, moderate, low, and very low) using the online software GRADEpro GDT [24] according to the Grading of Recommendation, Assessment, Development, and Evaluation (GRADE) approach [25].

## Results

### Search results

**Fig. 1:**
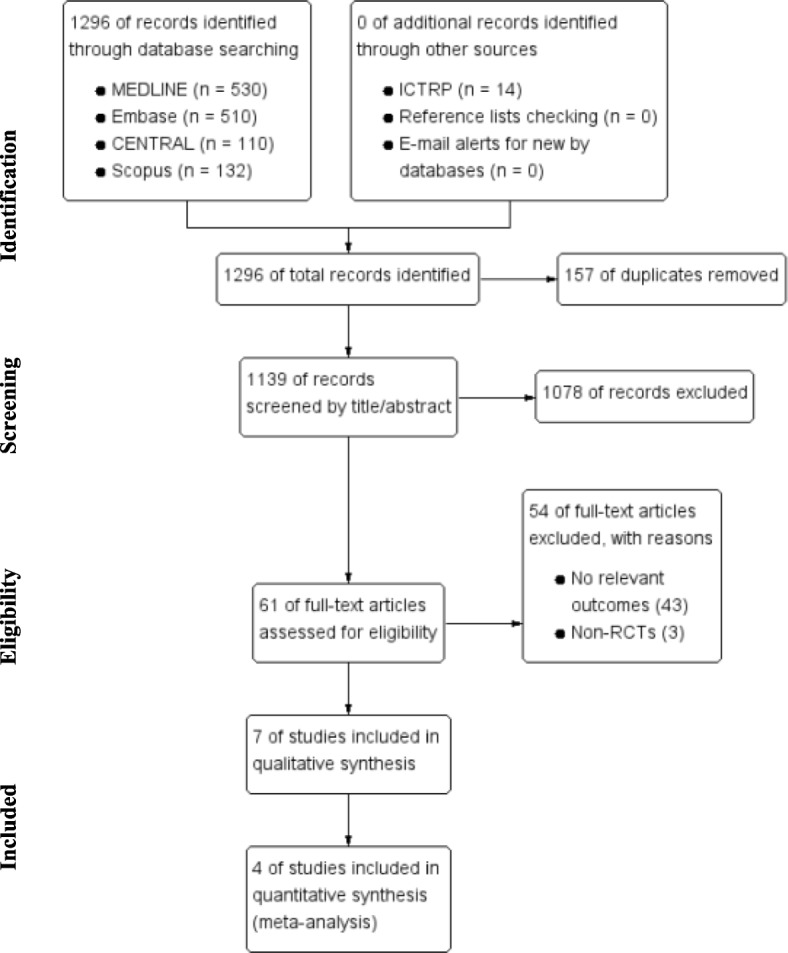
PRISMA flow diagram

The full details of the search results were summarized in Fig. 1. The initial search algorithm identified 1296 articles; 1139 studies remained after 157 duplicates were removed. Following the screening of titles and abstracts, 1078 studies were eliminated for not meeting the eligibility criteria. A total of 61 studies were retrieved for full-text reviewing; of these, 54 were excluded for not reporting the relevant outcome data or for being non-RCTs. Ultimately, seven studies were identified for the qualitative synthesis [13–15, 17, 26–28]; three studies [15, 26, 27] among those were excluded from the quantitative synthesis because the results were provided as figures only and the primary data could not be obtained.

### Study characteristics

The characteristics of the eligible studies are described in Table 1. We included a total of 7 trials with 316 patients (162 cases and 154 controls) in the qualitative synthesis. The trials included a small number of subjects each; mean age ranged from 40 to 70 years. Six studies were parallel RCTs [13, 14, 17, 26–28] and one was a crossover study [15]. The studies originated in four countries, including one from Belgium [14], two from Italy [13, 15], three from Japan [26–28], and one from Egypt [17]. From the 6 studies (276 patients) [13–15, 17, 27, 28] that reported the grading of HE at baseline, 244 (88%) patients were diagnosed with CHE and 13 (12%) with HE grade II. All patients in the included studies had cirrhosis and most were classified as CP class B (CP score 7–9 points) [29]. Furthermore, all patients were found to have zinc deficiency at baseline, but there was no statistically significant difference between the zinc and control groups in each trial. As for the intervention, four studies used combination therapy, including lactulose plus zinc supplementation [13, 17, 28] or branched-chain amino acid (BCAA) granules plus zinc sulfate [26]; while three studies used monotherapy treatment, such as zinc acetate [14, 27] or zinc sulfate [15]. The dose of zinc supplements varied between 50 mg and 600 mg per day. The treatment duration was less than 2 weeks in two studies [14, 15], and lasted for 6 months in five studies [13, 17, 26–28].

**Table 1:** Characteristics of the selected randomized controlled trials

Author, Year	Trial design	Country	N (M:F)	Age^a^ (yrs)	Etiology of cirrhosis (V/A/other)	HE grade (MHE/I/II)	Serum zinc levels^a^ (ug/dL)	CP score^a,b^/ classification	Intervention	Comparison	Treatment duration (days)
Reding, 1984 [14]	Randomized, double-blind, placebo-controlled	Belgium	Z: 10 (8:2)	Z: 52.1 ± 9.9	0/22/0	0/22/0	Z: 60.3 ± 17.9	Z: A/B/C = 1/8/1	Zinc acetate 600 mg/d	Placebo	7
			C: 12 (7:5)	C: 52.7 ± 13.4			C: 64.5 ± 21	C: A/B/C = 1/9/2			
Riggio, 1991 [15]	Double-blind crossover trial	Italy	10:5	47–71	6/5/4	3/10/2	29.9 ± 8.4	ND	Zinc sulfate 600 mg/d	Placebo	10
Bresci, 1993 [13]	Randomized, double-blind, placebo-controlled	Italy	Z: 46 (33:13)	Z: 51 ± 9	50/30/10	≤ grade I	Z: 50 ± 6	Z: A/B/C = 0/30/16	Zinc acetate 600 mg/d and lactulose	Lactulose 90 g/d	180
			C: 44 (23:21)	C: 49 ± 9			C: 52 ± 5	C: A/B/C = 0/35/9			
Hayashi, 2007 [26]	Randomized, double-blind, placebo-controlled	Japan	Z: 19 (10:9)	Z: 66.0 ± 9.9	38/0/2	ND	Z: 58.4 ± 9.2	ND	Zinc sulfate 200 or 600 mg/d^c^ and BCAA granules	BCAA granules	150–180
			C: 21 (13:8)	C: 65.1 ± 11.3			C: 60.2 ± 9				
Takuma, 2010 [28]	Randomized, unblinded, placebo-controlled	Japan	Z: 39 (17:22)	Z: 66.5 ± 5.7	58/13/8	0/49/30	Z: 48.9 ± 9.3	Z: A/B/C = 8/23/8	Polaprezinc 225 mg/d^d^ and standard therapy^e^	Standard therapy^e^ 30–60 mL/d	180
			C: 40 (23:17)	C: 66.5 ± 7.4			C: 51.6 ± 13.3	C: A/B/C = 7/26/7			
Katayama, 2014 [27]	Randomized, double-blind, placebo-controlled	Japan	Z: 7 (3:4)	Z: 64.3 ± 7.1	ND	≤ grade 1	Z: 55.1 ± 8.1	ND	Zinc acetate 150 mg/d	Placebo	90
			C: 5 (4:1)	C: 73.6 ± 8.4			C: 51.8 ± 8.3	ND			
Mousa, 2016 [17]	Randomized, double-blind, placebo-controlled	Egypt	Z: 31 (16/14)	Z: 54.5 ± 9.6	57/0/3	58/0/0	Z: 49.6 ± 11.2	Z: A/B/C = 6/22/3	Zinc gluconate 175 mg/d, Vit. A 50,000 IU, Vit. C 500 mg, Vit. E 100 mg, and lactulose	Lactulose 30–60 ml twice to three times a day	90
			C: 27 (15/12)	C: 55.8 ± 9.2			C: 46.9 ± 10.5	C: A/B/C = 4/20/3			

*M* male, *F* female, *N* number, *CP* Child-Pugh, *Z* zinc supplementation, *d* day, *C* control, *ND* no data, *HE* hepatic encephalopathy, *MHE* minimal hepatic encephalopathy, *V* viral, *A* alcoholic, *BZL* blood zinc level, *BCAA* Branched-chain amino acids

^a^Data reported as the mean ± standard deviation

^b^Cirrhosis staging reported as A: < 7; B: 7–9; and C: > 9

^c^The zinc sulfate dose was 600 mg/d when serum zinc levels of < 50 μg/dL and was 200 mg/d when zinc levels of 50–70 μg/dL

^d^Polaprezinc was composed of zinc 51 mg and L-carnosine 174 mg

^e^Standard therapy contained BCAA granules and lactulose 30–60 mL/d

### Quality assessment

Using the Cochrane Risk of Bias Tool, the methodological quality assessment of the seven selected trials were presented in the Additional file 3. Overall, the risk of bias was low or unclear for most items, but high for participants and personnel that were not blinded, in one study [28]. We were not able to comprehensively assess the risk of bias in five studies due to the lack of detailed information reported in the publication [13–15, 17, 26].

### Synthesis of results

#### Primary outcomes: psychometric tests

**Fig. 2:**
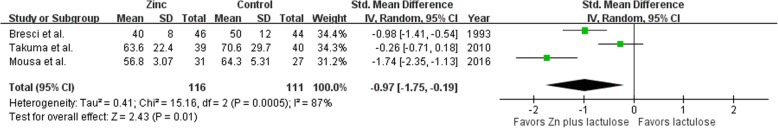
The NCT results of combination therapy of zinc supplementation and lactulose compared with only lactulose use

A total of four included studies reported the NCT results. Three studies [13, 17, 28], with a total of 227 patients comparing zinc supplementation plus lactulose versus lactulose alone, were included in the meta-analysis (Fig. 2). Pooled analysis showed that compared with the lactulose group, there was a significantly better performance on the NCT in the zinc supplementation plus lactulose group (SMD: -0.97; 95% CI − 1.75 to − 0.19, *P* = 0.01). However, a significantly considerable heterogeneity across the studies (*P* = 0.0005, I^2^ = 87%) was observed, but all studies showed the same direction of effect. We did not perform subgroup analysis due to the small number of studies.

**Fig. 3:**
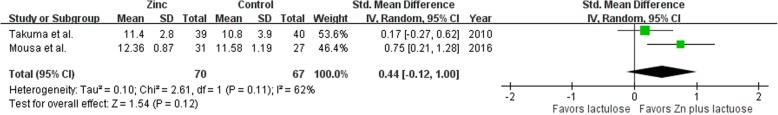
The DST results of combination therapy of zinc supplementation and lactulose compared with only lactulose use

Two studies [17, 28] with 137 patients, using the digital symbol test (DST) [30] as outcome, were combined in the meta-analysis (Fig. 3). There was a non-significant improvement in the DST results with combination of zinc supplementation and lactulose when compared with lactulose therapy alone. (SMD: 0.44; 95% CI − 0.12 to 1.00; *P* = 0.12; I^2^ = 62%).

In addition, there were two trials [14, 15] comparing short-term oral zinc supplementation to placebo in the NCT results. Reding et al. [14] showed that zinc supplementation administered orally for 1 week improved cirrhotic patients with HE grade I assessed by the NCT. Riggio et al. [15], who presented the contradictory results as figures only without the original data obtained, found no significant differences in NCT either during zinc or placebo administration. The treatment duration in both studies was too short to assess the efficacy of zinc supplementation, and was not appropriate to be pooled in our meta-analysis as well.

#### Secondary outcomes

**Fig. 4:**
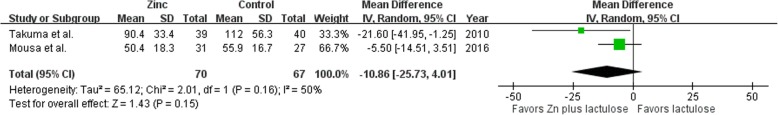
The serum ammonia levels of combination therapy of zinc supplementation and lactulose compared with only lactulose use

Two studies [17, 28] evaluating serum ammonia levels as outcome were pooled in the meta-analysis (Fig. 4). There was a non-significant reduction in serum ammonia levels in zinc supplementation plus lactulose group compared with lactulose group (MD − 10.86 μg/dL; 95% CI − 25.73 to 4.01 μg/dL; *P* = 0.15; I^2^ = 50%). No adverse events or hospitalization attributable to zinc supplementation were noted in any of the included trials.

Besides, three studies using plasma ammonia as an outcome measure were not pooled in the meta-analysis due to lack of raw data obtained. Hayashi et al. [26] reported that combination therapy of BCAA and zinc sulfate significantly decreased the post/pre-treatment change ratio in blood ammonia levels more than BCAA treatment alone in liver cirrhosis (0.87 ± 0.26 vs. 1.22 ± 0.38, *P* = 0.0033). Katayama et al. [27] showed that blood ammonia levels significantly decreased in the zinc group (*P* = 0.0114) compared with the placebo group. However, Riggio et al. [15] reported a conflicting result in that no significant difference was observed in serum ammonia levels between cirrhotic patients receiving zinc and those receiving placebo**.**

#### Summary of the findings

The estimates of the effect and the GRADE assessments for individual outcomes are presented in (Table 2). Overall, zinc supplementation has a large significant effect on improvement of NCT, while there was only a non-significant small effect on improvement of DST. The certainty of evidence was moderate for the NCT outcomes and very low for the others. All outcomes were downgraded because of the serious risk of bias from the lack of blinding in one study [28]. The DST and serum ammonia levels were downgraded because of serious imprecision of the 95% CI. The potential publication bias may be present for each outcome due to the limited number of included trials.

**Table 2:** GRADE assessment of the outcomes

Certainty assessment							№ of patients	Effect		Certainty
No. of studies	Study design	Risk of bias	Inconsistency	Indirectness	Imprecision	Other considerations	Zn	Placebo	Absolute (95% CI)	
Number connection test										
3	randomized trials	serious^a^	not serious	not serious	not serious	publication bias strongly suspected strong association^b^	116	111	SMD 0.97 lower (1.75 lower to 0.19 lower)	MODERATE
Digital symbol test										
2	randomized trials	serious^a^	not serious	not serious	serious^c^	publication bias strongly suspected strong association^b^	70	67	SMD 0.44 higher (0.12 lower to 1 higher)	VERY LOW
Serum ammonia lev										
2	randomized trials	serious^a^	not serious	not serious	serious^c^	publication bias strongly suspected strong association^b^	70	67	MD 10.86 lower (25.73 lower to 4.01 higher)	VERY LOW

*CI* Confidence interval, *SMD* Standardized mean difference, *MD* Mean difference

Explanations

^a^We downgraded by one level for serious risk of bias: one study was unblinded

^b^Publication bias was not assessed due to the limited numbers of included trials

^c^We downgraded by one level for serious imprecision: the wide confidence interval contains significant benefits and harm

## Discussion

In this meta-analysis, we demonstrated that additional zinc supplementation may have a significant effect on the performance of NCT when compared with lactulose therapy alone in cirrhotic patients with mild HE. In addition, although our study found that there was no statistically significant difference in the DST results and serum ammonia levels between paired groups, the direction of effect remained the same in each trial. This result was possibly due to the small sample size, causing inadequate statistical power to detect differences in the treatment effect.

It has been reported that approximately 30–80% of patients with cirrhosis have evidence of CHE, depending on the criteria used for the diagnosis and the study population [31, 32]. Patients with CHE often have abnormalities on psychometric testing, especially in domains of attention, motor speed and accuracy, and visuo-spatial coordination [33]. As a result, patients with CHE tend to have impairments in their daily function including driving and working capabilities [34–36], with an adverse impact on their quality of life [37]. Furthermore, CHE is associated with a higher risk of hospitalization, OHE development, and death [38].

The management of HE depends on its severity. However, compared with OHE, current evidence for the management of CHE is limited. Several controlled trials [39–41] have shown that lactulose improved the psychometric tests and health-related quality of life (HRQOL) in patients with CHE compared with placebo or no intervention. One meta-analysis [42] included nine RCTs showed that compared with placebo or no intervention, lactulose significantly improved the neuropsychological testing, prevented the progression to OHE, and improved HRQOL, but with no significant difference in the mortality and an increased risk of diarrhea. Although lactulose may have significant beneficial effects for patients with CHE, routine treatment for CHE is not recommended except on a case-by-case basis until further large, blinded studies prove its effectiveness [43]. Rifaximin may also have beneficial effects in the management of CHE. Sidhu et al. [44] reported that rifaximin significantly improved both cognitive function and HRQOL in patients with MHE. However, a cost-effectiveness analysis [45] concluded that rifaximin was not a cost-saving therapy for CHE at the current prices unless the monthly cost was less than $353. Probiotics may have a potential in the treatment of CHE [46]. One meta-analysis [47] that included 14 RCTs showed that compared to no treatment or placebo, the use of probiotic significantly improved MHE, decreased hospitalization rates, and prevented progression to OHE. However, probiotic is also not recommended as a therapeutic option for CHE because of its open-label nature, varying types, and doses [43].

Zinc is an essential cofactor in enzymatic reactions responsible for converting ammonia to urea via ornithine transcarbamylase in the liver and metabolizing ammonia to glutamine via glutamine synthetase in the skeletal muscle [48]. Zinc deficiency may impair both ammonia-reduction pathways and thus results in elevated ammonia levels, which is common in advanced cirrhosis [28]. Treatment with long-term oral zinc in patients with advanced cirrhosis has been shown to increase the formation of urea from amino acids [11]. Oral zinc supplementation is relatively well-tolerated with rare side effects of dyspepsia, copper deficiency (with long-term high dose use), and an interfering effect on quinolone or tetracycline antibiotics [49]. It is worthy of note that all included articles used zinc compounds as supplements, not elemental zinc. The maximum adult dose of elemental zinc is 40 mg daily [50]. Since different types of supplements contain various percentages of elemental zinc, it is warned that patients should follow the healthcare professionals’ instructions when receiving zinc supplements for medical treatment.

Our study is the first to investigate the effects of combination therapy of zinc supplementation and lactulose in cirrhotic patients with low grade HE (≤ grade II). We reported a larger significant effect (SMD = − 0.97%) in favor of additional zinc supplementation on the NCT results compared with lactulose alone than the previous meta-analysis (SMD = − 0.62%). Moreover, apart from the NST, we investigated different endpoints including DST, which was primarily used for assessing psychomotor speed and attention in cognitive function, as well as serum ammonia levels. Furthermore, we used the GRADE methodology to evaluate our confidence in the estimates of the treatment effect.

Some limitations still existed in our meta-analysis. First, we included small number of studies, which limited the population with HE of less than grade II. As such, we were unable to explore the validity of zinc supplementation to patients with more advanced HE. Secondly, there was some clinical heterogeneity in our study. This may be attributed to one trial [17] using concomitant therapies of zinc plus antioxidants and lactulose, which demonstrated a much better performance in the NCT compared with the lactulose therapy alone. Another heterogeneity may have resulted from variations in the grade of HE. It seemed that cirrhotic patients with a lower grade of HE (and in particular MHE) at baseline, were affected more by zinc supplementation than those with a higher grade of HE. Besides, the formulation, dose, or duration of zinc supplements varied remarkably across the studies, which may have led to subgroup differences. Finally, our study assessed the effect of zinc supplementation on cognitive functions only by two tests (NCT and DST), which were mainly involved in psychomotor speed and attention. We could not determine whether zinc supplementation has beneficial effects on different cognitive domains, including visuospatial perception, memory, executive function, language, and praxis. Therefore, future high-quality RCTs with a large sample size encompassing all degrees of HE with the evaluation of other psychometric or neuro-physiologic testing are warranted to further elucidate our findings.

## Conclusions

In conclusion, we found moderate certainty of evidence supporting the fact that the combination of zinc supplementation and lactulose over 3 to 6 months may improve the NCT in cirrhotic patients with low grade HE, compared with lactulose only. Recognition of this association may have implications in zinc supplementation usage as an adjuvant agent to treat patients with low grade HE.

## Additional files

Additional file 1: PRISMA checklist. (DOC 67 kb)

Additional file 2: Search terms and search strategy. (DOCX 34 kb)

Additional file 3: Methodological quality assessment of selected trials. (DOCX 16 kb)

## Data Availability

The datasets used and/or analyzed during the current study available from the corresponding author on reasonable request.

## Abbreviations

BCAA: Branched-chain amino acid
CHE: Covert hepatic encephalopathy
CI: Confidence interval
DST: Digit symbol test
GRADE: Grading of Recommendations, Assessment, Development, and Evaluation methodology
HE: Hepatic encephalopathy
HRQOL: Health-related quality of life
MD: Mean differences
MHE: Minimal hepatic encephalopathy
NCT: Number connection test
OHE: Overt hepatic encephalopathy
RCTs: Randomized-controlled trials
SMD: Standardized mean differences

## References

[1] Bustamante J, Rimola A, Ventura PJ, Navasa M, Cirera I, Reggiardo V, Rodes J (1999). Prognostic significance of hepatic encephalopathy in patients with cirrhosis. J Hepatol.

[2] Conn HO, Leevy CM, Vlahcevic ZR, Rodgers JB, Maddrey WC, Seeff L, Levy LL (1977). Comparison of lactulose and neomycin in the treatment of chronic portal-systemic encephalopathy. A double blind controlled trial. Gastroenterology..

[3] Patidar KR, Bajaj JS (2015). Covert and overt hepatic encephalopathy: diagnosis and management. Clin Gastroenterol Hepatol.

[4] Kappus MR, Bajaj JS (2012). Covert hepatic encephalopathy: not as minimal as you might think. Clin Gastroenterol Hepatol.

[5] Aldridge DR, Tranah EJ, Shawcross DL (2015). Pathogenesis of hepatic encephalopathy: role of ammonia and systemic inflammation. J Clin Exp Hepatol..

[6] Vilstrup H, Amodio P, Bajaj J, Cordoba J, Ferenci P, Mullen KD, Weissenborn K, Wong P (2014). Hepatic encephalopathy in chronic liver disease: 2014 practice guideline by the American Association for the Study of Liver Diseases and the European Association for the Study of the liver. Hepatology..

[7] Ferenci P, Herneth A, Steindl P (1996). Newer approaches to therapy of hepatic encephalopathy. Semin Liver Dis.

[8] Sharma P, Sharma BC (2013). Disaccharides in the treatment of hepatic encephalopathy. Metab Brain Dis.

[9] Plank LD, Gane EJ, Peng S, Muthu C, Mathur S, Gillanders L, McIlroy K, Donaghy AJ, McCall JL (2008). Nocturnal nutritional supplementation improves total body protein status of patients with liver cirrhosis: a randomized 12-month trial. Hepatology..

[10] Katayama K, Kawaguchi T, Shiraishi K, Ito T, Suzuki K, Koreeda C, Ohtake T, Iwasa M, Tokumoto Y, Endo R (2018). The prevalence and implication of zinc deficiency in patients with chronic liver disease. J Clin Med Res.

[11] Marchesini G, Fabbri A, Bianchi G, Brizi M, Zoli M (1996). Zinc supplementation and amino acid-nitrogen metabolism in patients with advanced cirrhosis. Hepatology..

[12] Loomba V, Pawar G, Dhar KL, Setia MS (1995). Serum zinc levels in hepatic encephalopathy. Indian J Gastroenterol.

[13] Bresci G, Parisi G, Banti S (1993). Management of hepatic encephalopathy with oral zinc supplementation: a long-term treatment. Eur J Med.

[14] Reding P, Duchateau J, Bataille C (1984). Oral zinc supplementation improves hepatic encephalopathy. Results of a randomised controlled trial. Lancet..

[15] Riggio O, Ariosto F, Merli M, Caschera M, Zullo A, Balducci G, Ziparo V, Pedretti G, Fiaccadori F, Bottari E (1991). Short-term oral zinc supplementation does not improve chronic hepatic encephalopathy. Results of a double-blind crossover trial. Dig Dis Sci.

[16] Chavez-Tapia NC, Cesar-Arce A, Barrientos-Gutierrez T, Villegas-Lopez FA, Mendez-Sanchez N, Uribe M (2013). A systematic review and meta-analysis of the use of oral zinc in the treatment of hepatic encephalopathy. Nutr J.

[17] Mousa N, Abdel-Razik A, Zaher A, Hamed M, Shiha G, Effat N, Elbaz S, Elhelaly R, Hafez M, El-Wakeel N (2016). The role of antioxidants and zinc in minimal hepatic encephalopathy: a randomized trial. Ther Adv Gastroenterol.

[18] Moher D, Liberati A, Tetzlaff J, Altman DG (2009). The PG. preferred reporting items for systematic reviews and meta-analyses: the PRISMA statement. PLoS Med.

[19] Conn HO (1977). Trailmaking and number-connection tests in the assessment of mental state in portal systemic encephalopathy. Am J Dig Dis.

[20] Higgins JPT, Green S. Cochrane Handbook for Systematic Reviews of Interventions. 2017; http://handbook.cochrane.org. Accessed 1 May 2018.

[21] Borenstein M, Hedges L, P. T. Higgins J, Rothstein H. Introduction to meta-analysis. Chichester: John Wiley & Sons; 2009.

[22] DerSimonian R, Laird N (1986). Meta-analysis in clinical trials. Control Clin Trials.

[23] Higgins JP, Thompson SG, Deeks JJ, Altman DG (2003). Measuring inconsistency in meta-analyses. BMJ..

[24] GRADEpro GDT (2015). GRADEpro guideline development tool [software].

[25] Guyatt GH, Oxman AD, Vist GE, Kunz R, Falck-Ytter Y, Alonso-Coello P, Schünemann HJ (2008). GRADE: an emerging consensus on rating quality of evidence and strength of recommendations. BMJ..

[26] Hayashi M, Ikezawa K, Ono A, Okabayashi S, Hayashi Y, Shimizu S, Mizuno T, Maeda K, Akasaka T, Naito M (2007). Evaluation of the effects of combination therapy with branched-chain amino acid and zinc supplements on nitrogen metabolism in liver cirrhosis. Hepatol Res.

[27] Katayama K, Saito M, Kawaguchi T, Endo R, Sawara K, Nishiguchi S, Kato A, Kohgo H, Suzuki K, Sakaida I (2014). Effect of zinc on liver cirrhosis with hyperammonemia: a preliminary randomized, placebo-controlled double-blind trial. Nutrition..

[28] Takuma Y, Nouso K, Makino Y, Hayashi M, Takahashi H (2010). Clinical trial: oral zinc in hepatic encephalopathy. Aliment Pharmacol Ther.

[29] Pugh RN, Murray-Lyon IM, Dawson JL, Pietroni MC, Williams R (1973). Transection of the oesophagus for bleeding oesophageal varices. Br J Surg.

[30] Joy S, Kaplan E, Fein D (2004). Speed and memory in the WAIS-III digit symbol—coding subtest across the adult lifespan. Arch Clin Neuropsychol.

[31] Maldonado-Garza HJ, Vazquez-Elizondo G, Gaytan-Torres JO, Flores-Rendon AR, Cardenas-Sandoval MG, Bosques-Padilla FJ (2011). Prevalence of minimal hepatic encephalopathy in cirrhotic patients. Ann Hepatol.

[32] Bale A, Pai CG, Shetty S, Balaraju G, Shetty A (2018). Prevalence of and factors associated with minimal hepatic encephalopathy in patients with cirrhosis of liver. J Clin Exp Hepatol.

[33] Nardone R, Taylor AC, Holler Y, Brigo F, Lochner P, Trinka E (2016). Minimal hepatic encephalopathy: a review. Neurosci Res.

[34] Wein C, Koch H, Popp B, Oehler G, Schauder P (2004). Minimal hepatic encephalopathy impairs fitness to drive. Hepatology..

[35] Bajaj JS, Hafeezullah M, Hoffmann RG, Varma RR, Franco J, Binion DG, Hammeke TA, Saeian K (2008). Navigation skill impairment: another dimension of the driving difficulties in minimal hepatic encephalopathy. Hepatology..

[36] Groeneweg M, Moerland W, Quero JC, Hop WC, Krabbe PF, Schalm SW (2000). Screening of subclinical hepatic encephalopathy. J Hepatol.

[37] Labenz C, Baron JS, Toenges G, Schattenberg JM, Nagel M, Sprinzl MF, Nguyen-Tat M, Zimmermann T, Huber Y, Marquardt JU (2018). Prospective evaluation of the impact of covert hepatic encephalopathy on quality of life and sleep in cirrhotic patients. Aliment Pharmacol Ther.

[38] Patidar KR, Thacker LR, Wade JB, Sterling RK, Sanyal AJ, Siddiqui MS, Matherly SC, Stravitz RT, Puri P, Luketic VA (2014). Covert hepatic encephalopathy is independently associated with poor survival and increased risk of hospitalization. Am J Gastroenterol.

[39] Watanabe A, Sakai T, Sato S, Imai F, Ohto M, Arakawa Y, Toda G, Kobayashi K, Muto Y, Tsujii T (1997). Clinical efficacy of lactulose in cirrhotic patients with and without subclinical hepatic encephalopathy. Hepatology..

[40] Sharma P, Sharma BC, Puri V, Sarin SK (2008). An open-label randomized controlled trial of lactulose and probiotics in the treatment of minimal hepatic encephalopathy. Eur J Gastroenterol Hepatol.

[41] Dhiman RK, Sawhney MS, Chawla YK, Das G, Ram S, Dilawari JB (2000). Efficacy of lactulose in cirrhotic patients with subclinical hepatic encephalopathy. Dig Dis Sci.

[42] Luo M, Li L, Lu CZ, Cao WK (2011). Clinical efficacy and safety of lactulose for minimal hepatic encephalopathy: a meta-analysis. Eur J Gastroenterol Hepatol.

[43] Vilstrup H, Amodio P, Bajaj J, Cordoba J, Ferenci P, Mullen KD, Weissenborn K, Wong P (2014). Hepatic encephalopathy in chronic liver disease: 2014 practice guideline by the European Association for the Study of the Liver and the American Association for the Study of Liver Diseases. J Hepatol..

[44] Sidhu SS, Goyal O, Mishra BP, Sood A, Chhina RS, Soni RK (2011). Rifaximin improves psychometric performance and health-related quality of life in patients with minimal hepatic encephalopathy (the RIME trial). Am J Gastroenterol.

[45] Bajaj JS, Pinkerton SD, Sanyal AJ, Heuman DM (2012). Diagnosis and treatment of minimal hepatic encephalopathy to prevent motor vehicle accidents: a cost-effectiveness analysis. Hepatology..

[46] Zhao LN, Yu T, Lan SY, Hou JT, Zhang ZZ, Wang SS, Liu FB (2015). Probiotics can improve the clinical outcomes of hepatic encephalopathy: an update meta-analysis. Clin Res Hepatol Gastroenterol.

[47] Saab S, Suraweera D, Au J, Saab EG, Alper TS, Tong MJ (2016). Probiotics are helpful in hepatic encephalopathy: a meta-analysis of randomized trials. Liver Int.

[48] Haussinger D, Schliess F (2008). Pathogenetic mechanisms of hepatic encephalopathy. Gut..

[49] Leise MD, Poterucha JJ, Kamath PS, Kim WR (2014). Management of hepatic encephalopathy in the hospital. Mayo Clin Proc.

[50] Dietary reference intakes for vitamin a, vitamin K, arsenic, boron, chromium, copper, iodine, Iron, Manganese, Molybdenum, Nickel, Silicon, Vanadium, and Zinc. Washington (DC): National Academies Press (US): Publishe; 2018. 25057538

