# Supplementary material for: Zinc supplementation in patients with cirrhosis and hepatic encephalopathy: a systematic review and meta-analysis
Source: Nutr J. 2019 Jul 6;18:34. doi: 10.1186/s12937-019-0461-3 (PMC6612144; doi:10.1186/s12937-019-0461-3)
Supplement: Supplementary file 2 — Search terms and search strategy. (DOCX 34 kb) [file 12937_2019_461_MOESM2_ESM.docx]

**Additional file 2. Search terms and search strategy.**

1. **Search Terms**

|  | Synonyms | Controlled vocabulary (MeSH Terms) |
| --- | --- | --- |
| **P** | encephalopath*  HE  MHE  hepatocerebral  hepatoencephalopath*  ammonemi*  ammonaemi*  hyperammonemi*  hyperammonaemi*  cirrho*  hepatopath*  (ammoni* or hypozincemi* or  hypozincaemi*)  and (liver or hepat*)  ((liver OR hepat*) ADJ4 (disease* OR insufficiency OR fibros* OR failure OR coma* OR stupor*)) | Liver Diseases (no exp)  Hepatic Insufficiency  Liver Failure  End Stage Liver Disease  Hepatic Encephalopathy  Liver Failure, Acute  Liver Cirrhosis  Liver |
| **I** | zinc  ZN  zincum | Zinc  Zinc Acetate  Zinc Sulfate  Zinc Compounds |

1. **Search Strategy**

| Database | # | Search syntax | Citations found |
| --- | --- | --- | --- |
| **MEDLINE (Ovid)** | 1 | (encephalopath* OR HE OR MHE OR hyperammonemi* OR hepatocerebral OR hepatoencephalopath* OR ammonemi* OR ammonaemi*or hyperammonaemi* OR cirrho* OR hepatopath* OR **(**(ammoni* OR hypozincemi* OR hypozincaemi*) AND (liver OR hepat*)**)** OR **(**(liver OR hepat*) ADJ4 (disease* OR insufficiency OR fibros* OR failure OR coma* OR stupor*)**)**).mp |  |
|  | 2 | exp Hepatic Encephalopathy/ OR Liver Diseases/ OR exp Hepatic Insufficiency/ OR exp Liver Cirrhosis/ OR exp Liver/ |  |
|  | 3 | (zinc OR ZN OR zincum).mp. |  |
|  | 4 | exp Zinc/ OR exp Zinc Acetate/ OR exp Zinc Sulfate/ OR exp Zinc Compounds/ |  |
|  | 5 | (1 OR 2) AND (3 OR 4) | **8154** |
|  | 6 | limit 5 to (meta analysis OR systematic reviews) | **SR: 28** |
|  | 7 | 5 AND (randomized controlled trial.pt. OR controlled clinical trial.pt. OR randomized.ab. OR randomised OR placebo.ab. OR drug therapy.fs. OR randomly.ab. OR trial.ab. OR groups.ab. not (exp animals/ not humans.sh.)) | **RCT: 530** |
| **EMBASE** | 1 | (encephalopath* OR HE OR MHE OR hyperammonemi* OR hepatocerebral OR hepatoencephalopath* OR ammonemi* OR ammonaemi*or hyperammonaemi* OR cirrho* OR hepatopath* OR **(**(ammoni* OR hypozincemi* OR hypozincaemi*) and (liver OR hepat*)**)** OR **(**(liver OR hepat*) NEAR/3 (disease* OR insufficiency OR fibros* OR failure OR coma* OR stupor*)**)**):ti,ab,de |  |
|  | 2 | 'Hepatic Encephalopathy'/exp OR 'Hyperammonemia'/exp OR 'Liver Disease'/de OR 'Liver Dysfunction'/exp OR 'Liver Cirrhosis'/exp OR 'Liver'/exp |  |
|  | 3 | (zinc OR ZN OR zincum):ti,ab,de |  |
|  | 4 | 'Zinc'/exp OR 'Zinc Acetate'/exp OR 'Zinc Sulfate'/exp OR 'Zinc Compounds'/exp OR 'Gluconate Zinc'/exp |  |
|  | 5 | (#1 OR #2) AND (#3 OR #4) AND [embase]/lim | **8592** |
|  | 6 | #5 AND ([systematic review]/lim OR [meta analysis]/lim) | **SR: 64** |
|  | 7 | #5 AND ('crossover procedure':de OR 'double-blind procedure':de OR 'randomized controlled trial':de OR 'single-blind procedure':de OR (random* OR factorial* OR crossover* OR cross NEXT/1 over* OR placebo* OR doubl* NEAR/1 blind* OR singl* NEAR/1 blind* OR assign* OR allocat* OR volunteer*):de,ab,ti) | **RCT: 510** |
| **Cochrane CENTRAL** | 1 | (encephalopath* OR HE OR MHE OR hyperammonemi* OR hepatocerebral OR hepatoencephalopath* OR ammonemi* OR ammonaemi*or hyperammonaemi* OR cirrho* OR hepatopath* OR **(**(ammoni* OR hypozincemi* OR hypozincaemi*) and (liver OR hepat*)**)** OR **(**(liver OR hepat*) NEAR3 (disease* OR insufficiency OR fibros* OR failure OR coma* OR stupor*)**)**)::ti,ab,kw |  |
|  | 2 | MeSH descriptor: [Hepatic Encephalopathy] explode all trees |  |
|  | 3 | MeSH descriptor: [Liver Diseases] this term only |  |
|  | 4 | MeSH descriptor: [Hepatic Insufficiency] explode all trees |  |
|  | 5 | MeSH descriptor: [Liver Cirrhosis] explode all trees |  |
|  | 6 | MeSH descriptor: [Liver] explode all trees |  |
|  | 7 | (zinc OR ZN OR zincum):ti,ab,kw |  |
|  | 8 | MeSH descriptor: [Zinc] explode all trees |  |
|  | 9 | MeSH descriptor: [Zinc Acetate] explode all trees |  |
|  | 10 | MeSH descriptor: [Zinc Sulfate] explode all trees |  |
|  | 11 | MeSH descriptor: [Zinc Compounds] explode all trees |  |
|  | 12 | (#1 OR #2 OR #3 OR #4 OR #5 OR #6) AND (#7 OR #8 OR #9 OR #10 OR #11) @Trials | **RCT: 110** |
| **4)**  **Scopus** | 1 | TITLE-ABS-KEY (encephalopath* OR HE OR MHE OR hyperammonemi* OR hepatocerebral OR hepatoencephalopath* OR ammonemi* OR ammonaemi*or hyperammonaemi* OR cirrho* OR hepatopath* OR **(**(ammoni* OR hypozincemi* OR hypozincaemi*) and (liver OR hepat*)**)** OR **(**(liver OR hepat*) W/3 (disease* OR insufficiency OR fibros* OR failure OR coma* OR stupor*)**)**) |  |
|  | 2 | TITLE-ABS-KEY (zinc OR ZN OR zincum) |  |
|  | 3 | #1 AND #2 | 323 |
|  | 4 | #3 AND TITLE-ABS-KEY (randomized OR placebo OR drug therapy OR randomly OR trial OR groups) | 140 |
|  | 5 | #4 AND ( EXCLUDE ( SUBJAREA , "VETE" ) OR EXCLUDE ( SUBJAREA , "AGRI" ) OR EXCLUDE ( SUBJAREA , "CENG" ) OR EXCLUDE ( SUBJAREA , "CHEM" ) OR EXCLUDE ( SUBJAREA , "ENVI" ) ) | **RCT: 134** |
| **4)**  **ICTRP** | 1 | (encephalopath* OR hyperammonemi* OR hepatocerebral OR hepatoencephalopath* OR ammoni* OR hyperammonaemi* OR cirrho* OR hepatopath* OR ((ammoni* OR hypozinc* OR disease* OR insufficiency OR fibros* OR failure OR coma* OR stupor*) and (liver OR hepat*))) @Condition  AND (zinc OR ZN OR zincum) @Intervention | **CT: 14** |
