# Supplementary material for: Zinc supplementation in patients with cirrhosis and hepatic encephalopathy: a systematic review and meta-analysis
Source: Nutr J. 2019 Jul 6;18:34. doi: 10.1186/s12937-019-0461-3 (PMC6612144; doi:10.1186/s12937-019-0461-3)
Supplement: Supplementary file 3 — Methodological quality assessment of selected trials. (DOCX 16 kb) [file 12937_2019_461_MOESM3_ESM.docx]

**Additional file 3. Methodological quality assessment of selected trials.**

| **Author** | **Sequence generation** | **Allocation concealment** | **Blinding of participants and personnel** | **Blinding of outcome assessment** | **Incomplete outcome data** | **Selective reporting bias** | **Other biases** |
| --- | --- | --- | --- | --- | --- | --- | --- |
| Reding et al. | Unclear | Unclear | Low | Unclear | Low | Low | Low |
| Riggio et al. | Low | Low | Low | Low | Low | Low | Unclear |
| Bresci et al. | Unclear | Unclear | Low | Unclear | Low | Low | Low |
| Hayashi et al. | Unclear | Unclear | Unclear | Unclear | Low | Low | Unclear |
| Takuma et al. | Low | Low | High | Low | Low | Low | Low |
| Katayama et al. | Low | Unclear | Low | Unclear | High | Low | Unclear |
| Mousa et al. | Unclear | Unclear | Low | Unclear | Low | Low | Low |
